# Supplementary material for: Host immunity increases Mycobacterium tuberculosis reliance on cytochrome bd oxidase
Source: PLoS Pathog. 2021 Jul 28;17(7):e1008911. doi: 10.1371/journal.ppat.1008911 (PMC8351954; doi:10.1371/journal.ppat.1008911)
Supplement: Attachment — Submitted filename: Response to Reviewers.docx [file ppat.1008911.s001.docx]

Reviewer #1: Elucidating the role of the alternate branch of the aerobic electron transport chain in mycobacteria terminating in cytochrome bd oxidase has been a topic of considerable interest particularly in light of the discovery of QcrB as a vulnerable TB drug target and subsequent clinical development of Q203 as a new drug candidate. While there is abundant evidence of bd oxidase upregulation in mycobacteria in response to disruption of the main cytochrome bc1-aa3-terminating branch of the ETC, the specific cellular function/s served by the bd oxidase in the physiology of Mtb has remained elusive. This problem has been further compounded by discrepant findings on the impact of inactivating mutations in the cydABDC gene cluster on growth and persistence of Mtb during mouse infection with one earlier study revealing a persistence defect in late-stage infection, but others showing no discernible effect.

In this manuscript, the authors re-visit this question and demonstrate a role for the bd oxidase in resisting the host adaptive immune response via an atypical IFNg-dependent mechanism which does not rely on reactive nitrogen or oxygen radicals to inhibit the bd oxidase, but is manifest instead at low pH. In a significant advance, they establish a role for the bd oxidase in respiration under acidic conditions and accordingly show potentiation of anti-TB activity of Q203 at low pH. This is a clearly written manuscript that describes some important results of general interest.

Reviewer #2: The cytochrome bd oxidase is a bacteria-specific terminal oxidase involved in microaerophilic respiration and resistance to oxidative stress in several bacteria, including Mycobacterium tuberculosis. In this article, the author reports on the involvement of the cytochrome bd oxidase in resistance to host immunity in macrophages and in a mouse model of tuberculosis infection. Using an H37Rv ΔcydC strain, a series of experiments was conducted to demonstrate the requirement of the bd oxidase for optimal growth at acidic pH, for viability in IFN-g activated macrophages (in a NOS2- and Cybb-independent manner) and for respiration at low pH. The article has some merits but is to some extent difficult to read and evaluate given the lack of experimental details, heavy reliance on data normalization and -on few occasions- issues with data analysis. The rational for using several statistical methods throughout the study should be explained.

Reviewer #3: The paper by Sassetti and colleagues reports the role of cytochrome bd (cyd) of M. tuberculosis in resisting the adaptive immune response. The molecular basis for this adaptation was the role of cytochrome bd in protecting M. tuberculosis from the acidic pH of the IFNγ-activated macrophages. The authors demonstrate that oxygen consumption by the cyd mutant is decreased at low pH because the bcc-aa3 proton-pumping complex is inhibited by an unknown mechanism. The manuscript is important because it again validates the respiratory oxidases as essential targets in TB drug development.
The paper is well written and the data presented in an easy to follow structure. However, the OCR work is a little disappointing as crucial pieces of information are missing that this referee requires to make a suitable recommendation.

**We thank the reviewers for their careful and constructive review of our work. The general concerns expressed above regarding data normalization and statistical analysis are answered in detail below. The suggestions regarding the OCR studies were particularly helpful, and stimulated a entirely new set of more informative studies.**

**Part II – Major Issues: Key Experiments Required for Acceptance**
Please use this section to detail the key new experiments or modifications of existing experiments that should be absolutely required to validate study conclusions.

Generally, there should be no more than 3 such required experiments or major modifications for a "Major Revision" recommendation. If more than 3 experiments are necessary to validate the study conclusions, then you are encouraged to recommend "Reject".

**Reviewer #1:**

1. Line 185-187 and Fig. 4C and D. These refer to a ΔcydAB mutant, however this strain is not described in the Experimental Methods (line 264). A complemented derivative of the cydAB (or cydA?) mutant used in the flux analysis experiments shown in Fig. 4C and 4D must be included to confirm that the effect observed is attributable specifically to abrogation of bd oxidase function.

**The reviewer points out a weakness of the previous version of this work, which has been remedied. The extracellular flux analysis experiments were repeated with the Δ*cydA* and Δcyd*A::ABDC* strains that are used throughout the paper.**

**Reviewer #2:**

Figure 1:

1. Line 120/121: is the statement that addition of IFN-g significantly reduced the number of cells harboring live H37Rv supported by statistics (Fig 1b)?

**Yes. The requested statistical analysis is provided in the panel, with details in the figure legend.**

2. Mycobacterial viability in macrophages was monitored using a GFP/RFP reporter system, or CFU determination. FACS was used to determine the number of macrophages containing live bacteria (GFP+/RFP+) or dead bacteria (GFP+). Is the approach able to detect a cell containing both live and dead bacteria ? if not, this limitation should be discussed.

**The flow cytometry assay does not provide the type of single-bacillus data that the reviewer refers to. This would only be possible by laborious microscopic assays. However, this reporter system has been shown to correlate with bacterial viability in previous work (PMID: 22980326) and our study, and provides a rapid and robust complement to CFU enumeration. These issues are now discussed in lines 125-129 using the following text, “The *Mtb* strains expressed a constitutively expressed GFP marker and an anhydrotetracycline (ATc)-inducible RFP marker. GFP intensity was used to estimate total infected cell number and ATc-induced RFP intensity served as a surrogate measure of the relative viability of the bacterial population in each infected macrophage, and has been show to correlate with CFU in this setting (23).”**

3. line 125. The statement that the viability recorded with the report genes system (Fig 1b) correlates with CFU determination (Fig 1c) is not totally accurate since some statistical differences observed with the reporter genes were not confirmed by CFU determination. Please comment.

**To be consistent, we reanalyzed the data using the same statistical test used to analyze the reporter Mtb data in panel 1B (One-way ANOVA with Sidak’s post-test to make the requested comparisons). The current statistical analysis supports the original statement that the reporter data correlates with CFU data.**

4. The use of the ANOVA with Sidak post-test to analyze panel b and c, and the Dunnett’s multiple comparisons test for c need to be justified. What was the requirement to use ANOVA and the need for the Sidak post-test. How many times were the experiments repeated. Statistic should be applied to compared other conditions in panel d (e.g. H37Rv unstimulated vs H37Rv IFN-g stimulated or DcydD vs DcydD:cydABDC – both IFN-g activated).

**These are important issues that were not described adequately in the original manuscript. This was remedied in the following ways:**

- **We have added a “Statistical Analysis” section to the Methods, which describes our rationale for choosing specific ANOVA post tests. This section states, “Statistical tests and the number of replicate experiments performed are noted in each figure legend. For ANOVA analyses, the specific post-test used in each figure was chosen based on the experimental design. Sidak’s post-test was used for comparing multiple pre-defined comparisons. Tukey’s post-test was used if all samples were compared to each other.” We hope the reviewer agrees that it is appropriate to choose post-tests based on experimental design.**
- **To be consistent for the analysis done in Figure 1B-C, we re-analyzed the data in panel D using a one-way ANOVA with Sidak’s post-test. In this case, we are comparing all stimulated strains to *ΔcydA* in the IFNg-stimulated condition.**
- **We disagree that we should apply statistical tests to additional conditions in panel D. In a perfect world with unlimited sample sizes, there may be some benefit to testing all samples against all others. However, this approach greatly limits statistical power. For this reason, it is appropriate to limit comparisons to those that are relevant to the specific hypothesis being tested.**

5. The need to normalize the results in the panel c & d should also be explained, why not presenting the CFU numbers instead of normalized values?
   If the results are presented “as percent survival relative to untreated”, what does the standard deviation reflect in the untreated control groups?

**All sample values were transformed based on the following formula, [sample value]/[mean of untreated]*100. The standard deviation for each sample is based on these transformed values. This transformation was performed to correct for input CFU values, which improves the visual representation of the data. “Untreated” values are provided in the legend, for full transparency.**

Figure 2:

6. Co-infection was an excellent idea to compare the fitness of the cydA-deficient strain in animals. Few experimental details should be added to support the findings: inoculum size, number of animals per group, how many times the experiments were repeated, statistical method used. The bacterial load early after infection (e.g. 24 hours) should be shown to ensure that the implementation of both strains was in a similar range. If those data are not available, the enumeration of the ratio cydA-deficient/H37Rv in the inoculum would address this concern.

**These aerosol infections were repeated twice with n=3 per genotype. We have added these details to the the figure legend. We also included Day 1 CFU, as requested, to show that both strains were delivered in at a similar dose (Figure 2C).**

7. A limitation of the study is the lack of one late time point to enhance the confidence that the differences (which are at best 5-fold reduction) at day 30 are robust.

- **We agree that this is a limitation of our infection experiments. However, we note that these competition studies integrate fitness differences throughout the infection, and it is difficult to imagine that they would be less robust at a later time point. Regardless, we have elaborated on this issue in the discussion, “While it is possible that another animal model or an extended infection period would discover a more pronounced growth defect, even a small or transient difference in bacterial fitness reflects an evolutionary advantage that could be responsible for maintaining the *cydABDC* operon in the Mtb genome” (Lines 245-248).**

8. Lanbo Shi (PNAS 2005) reported that a H37Rv cydC multiplied well in NOS2-/- mice, whereas this study suggests an attenuation phenotype. What could explain the difference with this previous report?

**The text of Shi *et al* states that “*cydC*-deficient bacteria continued to grow to log_10_ 7.6 to day 42 in inducible NO synthase-deficient (NOS2^–/–^) mice, at which time the animals died (data not shown)”. Bacterial growth/fitness was not compared to wild type, so it is unclear if the *cydC* mutant showed a relative growth defect and we are therefore unable to relate these observations to our data.**

Figure 3:

9. My main concern on this part of the work is the use of several assay formats to monitor growth. The assay in Fig 3a-b was done in 96 well plates (I assume the medium was 7H9-OADC-tyloxapol). This assay format may induce early oxygen depletion that could explain the requirement of the cyt-bd for optimum growth. The growth does not seem to be logarithmic, suggesting that the conditions are sub-optimum for growth.
10. To rule out the influence of oxygen, the growth kinetics should be repeated at a higher constant oxygen tension (for instance in roller bottles or under slow agitation) to confirm the results. Comparing the results from Fig 3a-b with Fig 3c is not possible since the complementation studied was performed in a different assay format, and at only one time point. Fig 3c should be repeated in the same format as in Fig 3a-b and growth should be recorded at regular time interval to derive a growth rate.
11. Growth rate should be calculated to confirm (using statistics) that the cydA KO multiplies at a slower rate at acidic pH. The pH of the culture broth media should be monitor overtime since the use of a phosphate buffer may not be appropriate to maintain the pH at 4.5.

**While we acknowledge these important issues, we consider the similar findings in multiple assay conditions to be a strength of this work. Regardless, we have performed a number of additional studies to address these concerns:**

- **To ensure that our plate-based assay reflected aerated culture conditions, we repeated the study in agitated cultures over multiple time points, as suggested. Even a modest reduction in pH to 6.2 produced a significant growth defect in ∆cydA bacteria, compared to wild type or complemented strains. These data are now provided in (Fig 3C).**
- **As requested, we calculated growth rate from OD data (Fig 3B). The reviewer may note that these growth rates are presented relative to the pH 7 condition because we found that the cydA mutant consistently grows slightly faster than H37Rv in this assay, an effect which we have noted in the paper (Lines 173-174). We thank the reviewer for this suggestion, as the relative effect of pH on wild type and mutant strains is quite clear in this analysis.**
- **The citrate-phosphate buffer used in this study was used based on its wide buffering range (3-8) and its previous use for Mtb culture (PMC2538620). We confirmed the stability of this buffer by measuring the pH of wild type and mutant cultures. These details are now clearly cited in the methods section (Lines 332-334).**

12. The macrophage experiments look convincing but should be confirmed (maybe in wt or NOS2-/- macrophages only) by CFU determination. It is a bit of as stretch to claim that the growth rescue of the cydA KO strain is a direct consequence of the increase in the pH of the phagosome: Bafilomycin A1 has multiple effects on the phagosome maturation that could explain the rescue. Please discuss the limitation of Bafilomycin A1.

**We agree with the reviewers concerns regarding nonspecific effects of Bafilomycin. While we thought these caveats were clear in the original manuscript, we have made sure to highlight them in the revised version. In every case where BAF if mentioned, we refer to it as an inhibitor of “acidification and maturation of the phagosome”, and we conclude that “the NO-independent inhibitory effect of IFNγ on bd oxidase-deficient Mtb could primarily be attributed to the environment encountered in the mature acidified phagosome.” The text referring to this issue in the discussion is unchanged, “While these data are consistent with a primary role for the *bd* oxidase in adaptation to low pH conditions, we note that inhibition of phagosome acidification can have pleiotropic effects on processes such as phagosome-lysosome fusion and autophagy.”**

**We respectfully ask the reviewer to reconsider whether additional CFU data are necessary. While we understand the reviewer’s desire for CFU enumeration in every infection study, we would argue that it is relatively standard to use reporters once they have been validated to reflect CFU in a specific setting, as we did in Figure 1. Our studies meet the standard of the field, consistent with a wide variety of papers that use validated reporters as surrogates for bacterial fitness (e.g. PMC3517204, PMC4976004,** [**PMC4908603**](http://www.ncbi.nlm.nih.gov/pmc/articles/pmc4908603/)**,** [**PMC7862720**](http://www.ncbi.nlm.nih.gov/pmc/articles/pmc7862720/)**,** [**PMC7050990**](http://www.ncbi.nlm.nih.gov/pmc/articles/pmc7050990/)**,** [**PMC6013021**](http://www.ncbi.nlm.nih.gov/pmc/articles/pmc6013021/)**,** [**PMC5526202**](http://www.ncbi.nlm.nih.gov/pmc/articles/pmc5526202/)**,** [**PMC5960073**](http://www.ncbi.nlm.nih.gov/pmc/articles/pmc5960073/)**,** [**PMC6329560**](http://www.ncbi.nlm.nih.gov/pmc/articles/pmc6329560/)**,** [**PMC5238961**](http://www.ncbi.nlm.nih.gov/pmc/articles/pmc5238961/)**,** [**PMC6231781**](http://www.ncbi.nlm.nih.gov/pmc/articles/pmc6231781/)**,** [**PMC6351102**](http://www.ncbi.nlm.nih.gov/pmc/articles/pmc6351102/)**). To further respond to this concern and to be as transparent as possible, we revised the relevant paragraph to clearly state that we use the “the live/dead reporter as a surrogate for bacterial fitness”, and we verified that we never imply an effect on “growth”.**

Figure 4:

**The following comments related to Figure 4 were particularly insightful and stimulated a new series of more informative studies. We will briefly respond to each point, below, and then describe the new studies.**

13. I have some difficulties understanding the design of the respirometry experiments. What seem to have been measured and discussed is not the effect of the pH on OCR, but the adaptive response after treatment with Q203.

Instead of the % OCR (that seems to indicate that the baseline respiratory rates were normalized), it would be more appropriate to present the data in absolute unit (pmol O2 consumed/min). In additional experiments, the bacteria should also be pre-incubated at pH 7.4 and 4.5 for 30 to 60 min before OCR recording (in absolute unit) to give a better reflection of the effect of low pH on mycobacterial respiration. Alternatively, OCR recording could be extended to few hours.

**Both the “adaptive response” to Q203 and the requested pre-incubation studies are informative, and are provided in the new Figure 4. The reviewer will note that two panels still report %OCR (normalized to the baseline OCR indicated in the figure) for clarity of presentation. The normalization is now clearly explained in the legend, so the reader can see this this represents a minor transformation of the data. When appropriate (4C) OCR is expressed in pmol/min, as suggested.**

14. The statement “At pH 4.5, Q203-treated cells displayed an even higher OCR than at neutral pH, potentially indicating further inhibition of the cytochrome bc1/aa3 complex and increased reliance on cytochrome bd” is not accurate. It is not the OCR that is higher at low pH, but the extend of the OCR deregulation induced by Q203, which is a phenomenon not fully understood. The use of bedaquiline as a control would be a good addition.

**These data have been replaced and the interpretation has been revised to specifically note that pH alters the adaptive response to Q203. We include BDQ as suggested, and it has a similar overall effect as Q203 – though the kinetics of the effects differ.**

15. The interpretation of the results obtained with the cydA-deficient strain (lines 186-188) is peculiar. The mutant is already in a medium at either pH of 7.4 or 4.5. Injecting few ul of the same medium at the same pH should not trigger any change in OCR. I do not really follow how this observation could indicate that the bc1-aa3 is preferentially reduced under these conditions (lines 189-190). Was this experiment repeated more than once?

**This study has been replaced, and the discussion revised to clearly compare OCR in wild type and cydA-deficient strains at different pH conditions. All experiments in figure 4 were performed twice, and this is noted in the legend.**

16. As presented and analyzed, the respiratory experiments do not seem to support the conclusion that Mtb reliance on the cyt-bd is higher a low pH.

**Figure 4 now shows completely new data that demonstrate three important points. First, the conditions used in our assay allow us to clearly differentiate OCR from either bd oxidase or *bc_1_/aa_3_* activity (4A). Secondly, the adaptive response to Q203 is entirely dependent on cydA, and is increased at low pH (4B). Finally, *bc_1_/aa_3_* dependent OCR is decreased at low pH (4C). Based on these data, we now conclude “the bd oxidase is functional, and even operates at increased levels, at acidic pH; whereas the bc1/aa3 is inhibited under these conditions.” (Lines 211-212).**

17. The observation that Q203 has a lower MIC at pH 5.5 is interesting. For a direct comparison between the OCR and the MIC results, it would be nice to run both experiments at a similar pH.

**We attempted to perform MIC assays at the lower pH used for OCR measurements, but were not able to detect sufficient growth under these conditions for accurate MIC determination.**

18. panel E. By default, it is a better practice to plot the raw OD600 values, what is the justification to normalize the values? Even if the bacteria multiply at a reduced rate at low pH, MIC50 can still be determined accurately. I would like to see few controls such as bedaquiline and rifampin to ensure that the shift in MIC is specific to Q203. To facilitate the experimental design, I would advise to use a microplate format (instead of the larger 10 ml volumes) and 2-fold drug dilution (8 points) to derive an accurate MIC50.

**Because H37Rv growth at pH 7 and pH 5.5 was very different, we prefer to retain the original data presentation for clarity. While the reviewer’s other suggestions are reasonable follow-up studies, both our data in figure 3 and the comments from the other reviewers indicate that the suggested static microplate-based assays with potentially variable oxygen concentrations would be suboptimal. Performing an extensive new study in a more laborious aerated cultures format is not warranted. The Q203 MIC study that we present is robust and reproducible, and testing additional drugs will not change this conclusion.**

19. There are few inconsistencies between the figure legends and the method section that need to be fixed. For instance, it is not clear if Q203 was used at 10 nM or at 900 nM in the respiratory experiments.

**These inconsistencies have been corrected and all figure legends have been updated. We thank the reviewer for the careful review.**

Reviewer #3:

1. Fig 1A – did OD600 mirror cell viability (CFU) given the culture would have been experiencing hypoxia at day 12?

**The only conclusions drawn from Figure 1A are that the ∆cydA strain grows similarly to wild type in aerated culture. While the reviewer’s question is an interesting one, answering it adequately would require a dedicated long-term study of hypoxia.**

2. 2. Did the authors perform an additional control for Fig. 1D by transforming CydABDC into the wild-type strain?

**To our knowledge it is not common practice to include such a control, so this strain was not made.**

3. Fig. 3: please report the growth rate as a function of pH – calculated from logscale plots. The cydA mutant looks to be growing faster than WT? What is the actual difference here? In each of the pH values tested what was the final pH of the culture measured at 5 h? Does the pH increase or remain relatively constant? Same comment for Fig. 3C – what was growth rate?

**Reviewer #2 had a similar concern; we now include growth rates calculations (Fig 3B) (See reviewer #2 comments 9-11). The reviewer is correct that the Δ*cydA* strain consistently grew slightly faster than Rv in 96 well plate assays, as is noted in the text. This effect is not seen in aerated cultures, which are included in Figure 3 to bolster our findings using multiple culture models.**

**The pH of wildtype and mutant cultures were measured 7 days after inculcation and remained relatively constant. Now described in the methods section “After a week in culture, the pH of the media was measured to ensure it was properly buffered. The pH 7 media was between pH 6.75-6.91, pH 6.2 media was between 6.23-6.30, and pH 5.7 media was between pH 5.60-5.70. There was no significant difference in media pH between H37Rv and Δ*cydA* cultures.”**

4. Fig. 3: What does cell survival look like as a function of external pH? Do the cells also die at acidic pH and if so what is the mechanism of cell death – the obvious explanation is a defect in intracellular pH homeostasis. Can the authors comment on this?

- **Figure 3 is focused on growth rate, and OD600 is a standard assay for this metric. We now provide new CFU data in Figure 5C demonstrating H37Rv viability was not impacted at pH 5.7.**

5. Fig. 4: to make sense of this figure I need to know what 100% OCR is for the WT and CydAB mutant – are the rates the same? I need to see the data so I can make sense of the interpretation. Same comment applies to OCR at pH 7.4 and 4.5 –for wild-type and cyd what are the actual rates for 100% OCR?

**Reviewer #2 had similar concerns (see points 13-16). These data and the associated discussion have been replaced in the revised manuscript. Two panels are presented as normalized OCR, and the method of normalization is presented in the legend so that it is clear that only minor data transformation was performed for clarity. The most relevant panel to this comment (4C) presents raw pmol O2/ min.**

6. Fig4E: why do the cells become more inhibited by Q203 at acidic pH if CydAB+ is still present? What does this data look like in the cyd mutant? Does Q203 (bacteriostatic) become bactericidal against the wild-type at acidic pH – this is an essential experiment.

**Multiple points:**

- **We are unable to answer the first question with certainty. We hypothesize that low pH is inhibiting the activity of the QCR system, making it more sensitive to Q203 inhibition. This hypothesis is used to introduce these data (lines 214-216).**
- **It is not possible to perform the Q203 MIC study with the ∆cydA mutant, as this strain does not grow at the relevant pH.**
- **Thank you for suggesting that we test for bactericidal activity. We found that Q203 is indeed bactericidal at low pH (Figure5B).**

7. Discussion page 9: why does low pH preferentially inhibit the bcc1-aa3 complex? You clearly show that the cyd mutant can still respire at acidic pH – need to know what the actual rate is in Fig. 4D to make any rational judgements.

**The new OCR data in Figure 4C should answer this question. Steady-state OCR in the ∆cydA mutant (which is bcc1-aa3 dependent) is** **reduced by ~3-fold at low pH. We can only speculate on the mechanism underlying this effect, and we do so in the discussion (lines 265-274).**

8. Lines 78-80: cyd mutants of M. smegmatis are also hypersusceptible to bedaquiline and cydAB is induced by BDQ – cite Hards et al. J Antimicrob Chemother 2015; 70: 2028–2037 (reference 35 covers M. tb only)

- **We have included this citation in the discussion (Line 279- Reference 41).**

9. Paper by the group of Rubin (ref 27) needs to be given a little more kudos for identifying the essential role for the cyd operon at acidic pH. Add to introduction.

- **This citation is now included in introduction, results, and discussion sections.**

**Part III – Minor Issues: Editorial and Data Presentation Modifications**
Please use this section for editorial suggestions as well as relatively minor modifications of existing data that would enhance clarity.

Reviewer #1:

1. Figure 1B and 1C, and text describing the experiments shown in this figure (lines 111-133). These figures show the statistical significance of comparisons between WT and ΔcydA strains. However, the more informative comparisons are between WT or mutant +/- IFNγ rather than between WT vs. mutant under a given condition (+/- IFNγ). Therefore, does either assay (Live/Dead or CFU) support the claim that the: “In wildtype BMDMs, addition of IFNg significantly reduced the number of cells harbouring live H37Rv and ΔcydA bacteria (Figure 1B)”, and if so, at what level of significance? Likewise, for the statement: “The CFU assay also showed that IFNγ treatment reduced the viability of H37Rv in a Nos2-dependent and Cybb-independent manner….”.

**All of the requested comparisons are made in the revised figure.**

2. Line 82. Dhar & McKinney (PMC2901468) identified a transposon mutant in cydC in a screen for Mtb mutants that show accelerated clearance by INH in mice in a manner “dependent on environmental changes imposed by the IFN-γ-mediated immune response”. This paper is highly relevant to the work described in this study and must therefore be cited, and its implications for the work reported here discussed.

**We added this citation to the discussion section of the paper. (Line 241-Reference 33)**

3. The authors attribute their ability to discern a persistence defect in IFNγ-competent mice to the fact that the experiment was done as a co-infection of WT and cydA mutant strains; even then, the persistence defect at day 30 is very modest (Fig. 2B). Is this due to the fact that in a competition assay, the cydA mutant has a competitive disadvantage against WT owing to an impaired ability to utilise (limited) oxygen during chronic infection? To broaden its appeal, the manuscript would also benefit from a fuller explanation of the implications of the findings for Mtb pathogenesis, including in the context of all the published literature. For example, what do the results reported here mean in terms of acidic (and hypoxic?) microenvironments in different mouse infection models? How does this differ in other animal models, in particular, NHPs? Can the authors speculate on why treatment of Mtb-infected marmosets with a bc1-aa3 inhibitor gave rise to cavitation, as reported by Beites et al.?

**We elaborate on some of these points in the discussion section. However, we are wary of wandering too far outside the scope of the study. We did not conduct any *in vitro* hypoxia experiments and it is difficult to dissociate the effects of oxygen availability and pH in the mouse model. It is even more difficult to speculate how our results relate to pathogenesis in other more complex models of infection (i.e. NHP), or why bc1-aa3 inhibition is associated with cavitation. In the end, we are only comfortable with the newly expanded speculation in lines 245-248.**

4. Line 79: Kana et al. (PMC95555) were the first to demonstrate induction of the cydABDC operon and a competitive growth disadvantage of a bd oxidase of M. smegmatis under hypoxia. This reference should be cited.

**We have included this citation (Line 92).**

Reviewer #2: -

Reviewer #3: (No Response)
